# A Rapid Chemiluminescence Assay for Measurement of Folate in Small Volumes of Breast Milk

**DOI:** 10.3390/molecules24152730

**Published:** 2019-07-27

**Authors:** Laurence Guignard, Chiara Nembrini, Julie Moulin, Karine Meisser, Irma Silva-Zolezzi, Jürgen Kratzsch, Mandy Vogel, Wieland Kiess, Erik Eckhardt

**Affiliations:** 1 Nestlé Research, Société des Produits Nestlé, 1000 Lausanne, Switzerland; 2 Institute of Laboratory Medicine, Clinical Chemistry and Molecular Diagnostics, University of Leipzig, 04103 Leipzig, Germany; 3 Hospital for Children and Adolescents and Center of Pediatric Research, University of Leipzig, 04103 Leipzig, Germany

**Keywords:** chemiluminescence, folate, breast milk

## Abstract

Early life exposure to folate has long lasting effects on development and health. Newborns obtain part of their folate from maternal milk. Studies on health effects of milk folate require rapid, affordable and reliable measurements in large numbers of samples from cohort studies. Recently, a competitive chemiluminescence assay for quantification of folate has become available for automated diagnostic measurement of folate in human serum or plasma. We tested if this method (“FOLA” from Siemens Healthcare) could also be used for human milk. To minimize interference and matrix effects, samples had to be skimmed, diluted seven times with demineralized water, and heated for 5 min at 90 °C. Folate could thus be measured in a linear range between 8.4 and 111.7 nM, with recoveries for the most relevant form, 5-methyltetrahydrofolate (5-MeTHF), of 96%–107%. Results were comparable to those with a recently validated Liquid Chromatography/Mass Spectrometry method (Y = 0.998X − 0.2; R^2^ = 0.807). The FOLA method was subsequently used for samples from the LIFE Child cohort in Germany, providing first data of breast milk folate in this country (range: 6.2–100.7 nM). This technique could indeed prove useful for large cohorts with multiple samplings.

## 1. Introduction

Folate plays essential roles in key cellular processes such as DNA methylation and cellular synthesis of nucleic- and amino acids [1]. Folate deficiency in utero leads to increased risk of fetal neural tube defects [2] and possibly other pathologies, such as childhood leukemia [3]. Pregnant women are therefore encouraged to eat folate-rich foods and take supplements with a synthetic form of folate, called folic acid. Several countries, including most of the Americas, have also implemented fortification strategies, with folic acid being added to wheat- or corn flour. In most of Europe, however, fortification has not been implemented.

Short or long-term health effects of postnatal folate intake are less known. Exclusively breast-fed infants derive their folate from breast milk (BM). The impact of maternal folate intake, being through diet, fortified food or supplementation on breast milk folate levels, remains controversial [4,5] and folic acid supplementation could lead to excess milk levels of folate with unknown health consequences. The adequate intake of folate for infants between 0 and 12 months is currently set at 66–80 µg/day [6] and is calculated based on an average breast milk folate concentration of 193 nM (85 µg/L) [5]. However, accurately assessing breast milk folate levels is not trivial [7] due to the presence of various substances in milk which can interfere with analytical methods and to the instability of 5-methyltetrahydrofolate (5-MeTHF), the most biologically relevant form of folate [7].

Up to now, most published breast milk folate data have been obtained with microbiological growth assays, in which samples are incubated with bacterial strains that rely on folate for growth (typically a Lactobacillus). Assays have been optimized to reduce matrix effects, such as trienzyme pretreatment [8] including deconjugase to free folate from its glutamate units and α-amylase and protease to release folate from the breast milk matrix. This treatment has not always been applied, making it difficult to compare published data. Moreover, results of microbiological growth assays may vary depending on the type of micro-organism [9]. The assays can be time consuming and error prone and are not ideal for large-scale screening of folate in large numbers of samples.

Some studies have used high performance liquid chromatography (HPLC) to quantify folates in breast milk [10,11,12,13]. With this technique, typically 5-MeTHF and folic acid are measured, while bacteriological assays likely quantify a larger range of folate forms (including mono- and polyglutamates) but without distinguishing individual forms. When the same samples are directly compared with both techniques, the results may differ, with HPLC typically yielding lower values [11]. This probably indicates that breast milk contains several folate forms, most of which may be detected in total by growth assays, whereas chromatography only measures forms for which the method has been calibrated. A study in Japan with HPLC reported amongst the highest breast milk folate levels in the literature [12], although there may have been methodological issues in that study [7].

Mother-child cohorts, such as the LIFE-Child cohort in Leipzig, Germany [14], can help shed light onto the relation between maternal nutritional status, breast milk composition, and infants’ health. Large-scale analyses of multiple samples of which often only small volumes are available would benefit from methodology that is less labor intensive than microbiological assays or HPLC. We therefore aimed to use a recently introduced clinical diagnostic assay originally developed for plasma and serum folate; namely the FOLA method. This method is based on competitive displacement of a folate form pre-bound to immobilized folate binding protein (FBP), a protein with strong affinity for most folate forms including folic acid [15]. In contrast to serum or plasma samples, which are directly injected into the auto-analyzer without pre-treatment, we describe at present that breast milk samples have to be pre-treated to reduce interference. For validation purposes, we compared values with those obtained in parallel using a recently validated LC-MS based methodology developed in-house. [10]

## 2. Results

### 2.1. Sample Pre-Treatment for Recovery and Measurement Optimization

Initial comparisons between full and skimmed milk revealed the need to use the latter because of interference by the fat phase with the assay resulting in irreproducible results (data not shown). Consequently, we decided to continue with skimmed milk only. Preliminary results revealed the absence of matrix effects upon serial dilutions, although recovery after spiking with a known amount of 5-MeTHF was <50% (not shown). We hypothesized that this was due to residual folate-binding capacity in the breast milk samples that could not be denatured by the heating step in the autoanalyzer. We therefore tested the minimum length of time needed to heat undiluted skimmed milk samples at 90 °C to maximize apparent folate measures by the autoanalyzer and observed that after 5 min maximum values were reached. Levels remained constant until 30 min, whereupon the folate signal was significantly lost (not shown). Thus, a 5 min heating step at 90 °C of the sample prior to the heating within the instrument was considered the optimal time to release maximum folate reactivity from any FBP without decreasing the viability of the released folate. However, recoveries were now in excess of 120%. We were unable to ascribe this additional signal to release from FBP of a possibly more potent folate form than 5-MeTHF or to release or generation of another molecule that causes a-specific signals during the assay. However, when heated samples had previously been diluted between six and seven times, recoveries of spiked 5-MeTHF reached 100% regardless of whether spiking occurred before or after the heating step. For method validation and any further analysis of unknown samples it was therefore decided to use skimmed milk, diluted 7-fold in water, followed by 5 min heating at 90 °C.

### 2.2. Analytical Range and Method Performance

**Figure 1:**
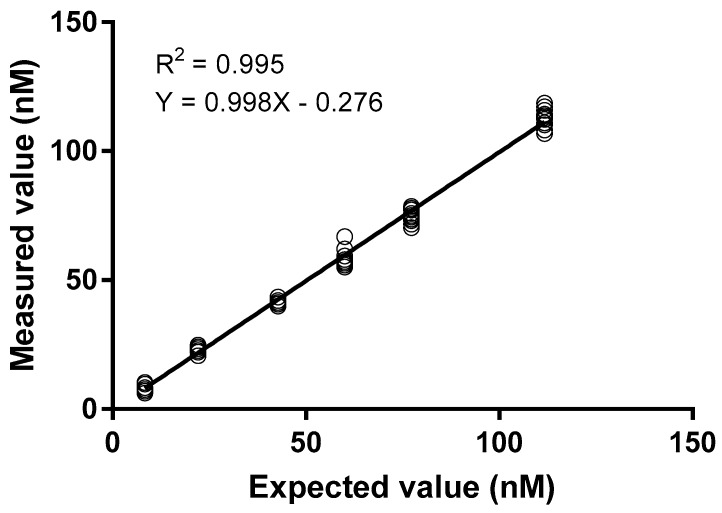
Linearity of folate measurements in spiking experiments show lower and upper levels of quantification of 8.4 and 111.7 nM, respectively. A pool of skimmed breast milk with folate levels at the lower limit of quantification (LLOQ) was thereto spiked at 5 different levels with the folate stock solution, diluted 7-times and then heated for 5 min at 90 °C (see Methods section for details). At each of these 6 levels, 6 measures were performed on 3 different days.

Method development and validation were performed with a pool of 10 skimmed breast milk samples containing the lowest folate levels. These had been identified by screening all samples from the Swiss cohort (see Methods section) after sample pretreatments described in 2.1. Because our sample pre-treatment included a 7-fold dilution step, we aimed to prepare a pool with folate values approaching 7-fold the lower limit of quantification (LLOQ) of the plasma FOLA assay (1.1 nM for undiluted plasma according to the manufacturer’s instructions, thus corresponding with a breast milk folate concentration of minimally 7 × 1.1 = 7.7 nM). The said pool appeared to have a final (i.e., dilution- corrected) concentration of 8.4 nM which was thus set as the LLOQ of the assay. The upper level of quantification (ULOQ) of the FOLA assay is 45.3 nM for undiluted plasma samples, which would set the theoretical upper level of breast milk at 317.1 nM. However, because the corrected maximum value observed in all tested breast milk samples was 120 nM, we decided to aim for a ULOQ at this value. The pooled breast milk (containing 8.4 nM folate) was therefore spiked with five concentrations of increasing amounts of folate (5.4, 26.0, 43.2, 60.5, 95.0 nM) to test linearity between 8.4 nM and 120 nM. Each sample and concentration were measured multiple times over multiple days while introducing operator and instrument variability. As shown in Figure 1, the method allowed for measurement of milk folate in a linear range between 8.4 and 111.7 nM.

The performance characteristics of the method, reflecting trueness, repeatability and reproducibility are outlined in Table 1.

As can be seen in the table, the performance characteristics of the method comply with standards set forth by the European Medicines Agency and with the values we aimed for as described in the methods section.

### 2.3. Comparison of Results with a LC-MS/MS Method

**Figure 2:**
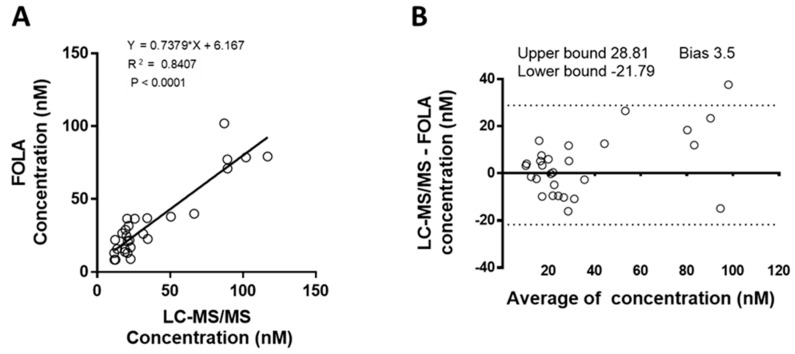
Correlation between FOLA and LC-MS/MS measurement. A series of 28 samples was measured with the new method and results were compared with measurements with an LC-MS/MS method. The chemiluminescent method measures all folate forms, while for the LC-MS/MS method, the sum of 5-methyl tetrahydrofolate (the main natural form) and folic acid (the synthetic form) is represented. Panel **A** shows the correlation between both methods, which is further illustrated by the Bland Altman plot in Panel **B**.

A previous report suggested that microbiological assays might lead to measurement of overestimated folate levels in breast milk [11]. We thus chose instead to compare the presented method with an LC-MS/MS method recently developed in house [10]. It is important to notice that this methodology detects and quantifies two major folate forms with vitamin B_9_ activity, namely 5-MeTHF and folic acid. Vitamin B_9_ concentration is therefore reported as the sum of 5-MeTHF and folic acid. In contrast, the FOLA assay detects any form of folate that binds FBP. The LC-MS/MS method quantifies 5-MeTHF with an accuracy of 99% with an inter-day precision of 4.9 percent. For folic acid, accuracy is 105.8% [10]. A series of 28 randomly selected breast milk samples was analyzed with our FOLA method and with the LC-MS/MS methodology in parallel. As shown in Figure 2, there was good correlation between values obtained by both methods (R^2^ = 0.8407; *p* < 0.0001).

### 2.4. Folate Stability in Breast Milk Samples

**Figure 3:**
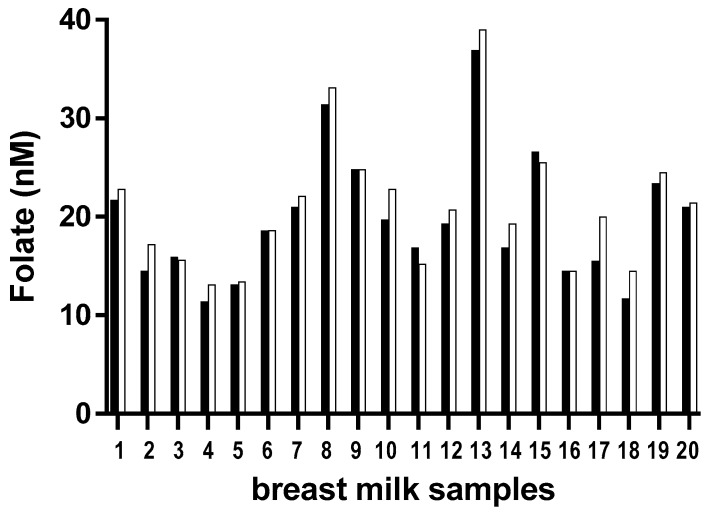
Apparent stability of folate levels in breast milk during prolonged storage (18 months at −80°C) The same 20 breast milk samples were measured in November 2015; black bars) and in May 2017 (open bars) with the modified FOLA method described in this manuscript.

Breast milk samples of observational cohorts are often collected and stored over longer periods of time. It is thus important to consider the stability of components such as folate in this particular matrix which might influence the decision of analyzing the samples as they are collected as opposed to at the very end of a study. We used our rapid method to test the stability of folate in 20 of the samples used for method validation. Samples were measured first at the time of collection in November 2015 and again in May 2017 after 18 months storage. Our results show that folate levels remained stable over this period (Figure 3).

### 2.5. Folate Levels in Milk Samples from the Life Cohort

We validated the FOLA method for breast milk with the aim of facilitating folate analysis in this matrix in future studies. At the same time, past and current studies in which breast milk samples were collected could also take advantage of this method, knowing that folate appeared to be stable over a period of 18 months (Figure 3). We thus set-out to test folate levels in 549 breast milk samples from the still recruiting LIFE Child mother-child cohort, obtained at 3, 6 or 12 months of lactation [14]. Only seven samples were found to have levels exceeding the method’s ULOQ and were thus excluded rather than re-measuring after an additional dilution. As shown in Table 2a, folate levels remained constant during lactation. Table 2b further summarizes the characteristics of participants providing the milk samples.

## 3. Discussion

This study describes the development and validation of a rapid and easily implementable method for the measurement of folate in a small volume of human breast milk. Due to the automated procedure and pre-treatment, the performance of our method proved to be acceptable in terms of trueness, reproducibility, repeatability, and other parameters (Table 1). The method could be used to measure breast milk folate immediately after obtaining a sample and could be performed in clinical centers equipped with a comparable clinical autoanalyzer, thus obviating the need for special laboratories with laminar flow hoods for bacteriological analyses or laboratories with sophisticated LC-MS/MS equipment. Moreover, the procedure could be implemented with minimal training. Since the method is fast (including skimming, results can be obtained within 1 h, with each additional sample only adding a few minutes of time), several hundred of samples can be measured in a matter of days.

However, there are possible shortcomings of this method, which are inherent to any method attempting to measure various biological relevant forms in a complex and highly variable biological matrix. First, we only used 5-MeTHF as an internal standard, whereas one cannot exclude that other bioactive forms exist in breast milk, such as folic acid found in supplemented food. Indeed, results with LC-MS/MS suggest that this form could be present in significant amounts in breast milk [10]. Moreover, we were unable to identify the reason for and nature of an unexpected small increase in folate signal when undiluted samples were heated to denature FBP. Selectivity of the method for folate in breast milk may thus not be as good as for plasma for which the FOLA method was originally designed, due to possibly larger variations in interfering substances in this matrix. Nevertheless, recovery of folate from spiked milk samples was good (Table 1). Another shortcoming could be that the ULOQ set for the method may prove to be somewhat low for analysis of milk samples from cohorts from countries with folic acid supplementation, in which folate levels may be higher than in the LIFE cohort.

One could also argue that the comparison method is not currently accepted as being the gold standard. This currently remains the microbiological assay, even though it may lead to an overestimation of folate levels as illustrated for example by the marked differences reported when samples were analyzed with a microbiological assay and, in parallel, with an HPLC-based method [11]. Indeed, LC-based analysis, when properly implemented, would be highly accurate and would allow for simultaneous qualitative and quantitative assessment of relevant folate species. With this in mind, we compared results obtained with the FOLA method with a recently developed and validated high quality LC-MS/MS methodology for measurement of water-soluble vitamins in breast milk [10]. Considering that LC-MS/MS measures only 5-MeTHF and folic acid, while our assay can in principle measure all folate species binding to FBP, we found a good correlation between the results obtained, strengthening our confidence in both methods (Figure 2). Nevertheless, the fact that the FOLA method showed lower values than the LC-MS/MS method in some samples while, in theory, it should detect more folate activity than the two main forms detected with LC-MS/MS illustrates that additional matrix effects cannot be ruled out in some milk samples. This illustrates the tremendous challenge one faces in developing a method for accurate detection of multiple forms of a vitamin in a series of biological fluids with considerable variation in composition.

The fact that both methods showed reasonable agreement in terms of total folate content while the FOLA method required skimming suggests that the skimming procedure does not significantly alter folate levels. This is not unexpected since folate is water soluble and should not enter the lipid phase of the milk when it is removed. However, inherently, by removing the small volume of fat from the milk, one slightly alters the concentration of water-soluble substances. Future tests should reveal if this small imprecision could be further reduced by diluting the milk prior to skimming, which would also reduce loss of precious milk volumes during skimming.

Currently available literature mentions breast milk folate levels that cover a rather wide range of values, from 11.2 to 320 nM (Table 3), and most publications report levels well in excess of the average values from the LIFE Child cohort reported in this study, which ranged from 27.2 to 34.7 nM depending on age of sampling.

These levels are also considerably lower than the breast milk concentration of 193 nM used to calculate adequate folate intake for infants in the first year of life [5]. However, most data had been obtained from cohorts in countries with established fortification strategies, suggesting that folic acid from fortified foods may be secreted into maternal milk either as folic acid or after conversion into a more bioactive form. In contrast, to our knowledge our study is the first to measure folate in more than 500 breast milk clinical study samples at different time points in subjects in continental Europe, more specifically Germany, where mandatory folic acid food fortification is not implemented. In fact, our values are closer to those reported in an earlier study in Nigeria in the absence of food fortification [16]. With the exception of this older study, our study may well be the only recent report determining breast milk folate in countries without mandatory folate fortification, also indicating that in its absence breast milk levels might be on the lower range.

The scientific evidence for the impact of folate maternal intake and corresponding breast milk levels is inconsistent [4,5]. Based on our results of the German LIFE cohort, it could be speculated that a long-term fortification strategy may lead to sustained higher levels of folate in breast milk. Importantly, the contribution of food fortification towards folate breast milk levels has not been directly addressed to date; further research comparing breast milk samples of countries with and without fortification policies, using the same folate analytical method, is warranted. Of note, some samples for the LIFE cohort did have higher levels of folate, which could still be the result of folate supplement intake by the donors.

Overall, folate breast milk levels in the German cohort seem to remain stable throughout the course of lactation, confirming previous observations (Table 2). Fortification strategies may thus be critical to ensure high breast milk folate levels from birth, as well as to maintain intact folate maternal storages, a role already suggested for folate supplementation [5].

In conclusion, we believe our method could enable more reliable comparisons of breast milk folate levels across different cohorts, thus helping reveal the impact of maternal diet, governmental fortification programs, and folic acid supplementation policies across different geographical areas. It should also allow to more reliably assess how breast milk folate levels relate to health in the infant or whether excess folic acid supplementation during lactation could have unexpected health consequences. Ultimately, this knowledge can be used to provide nutritional recommendations for folate intake in lactating mothers and infants.

## 4. Materials and Methods

### 4.1. Standards and Controls

Plasma control samples (Lyphochek Immunoassay Control PLUS) with 3 different precisely known folate concentrations for instrument calibration were purchased from Bio-Rad Laboratories (Cressier, Switzerland). (6*R*,*S*)-5-methyl-5,6,7,8-tetrahydrofolic acid (5-MeTHF), for spiking and recovery experiments, was purchased from Schircks Laboratories (Jona, Switzerland). A stock solution (10 nM) from accurately weighed 5-MeTHF was prepared. The solvent for 5-MeTHF consisted of three volumes methanol (Merck, Schaffhausen, Switzerland) and 1 volume water with a final buffer concentration of 2.49 mM ammonium acetate, 142 nM ascorbic acid, and 32.4 nM dithioerythritol (all from Sigma-Aldrich, Buchs, Switzerland) [10]. The 5-MeTHF standard solution was kept at −20 °C and diluted with water on each analysis day to a final concentration of 100 nM. The concentration of 5-MeTHF in this stock solution, determined with the chemiluminescent assay, remained constant over 6 months.

### 4.2. Breast Milk Samples

Breast milk samples used for method validation and subsequent comparison of the method with the LC-MS/MS method were obtained from healthy volunteers of the area of Lausanne, Switzerland, after written consent had been obtained using a form and protocol approved by the local ethical review board. The folate method was also used for the analysis of milk samples from a large cohort in Germany, the ‘Leipzig Research Centre for Civilization Diseases (LIFE) Child Study’ [14]. From this cohort, samples had been obtained at 3, 6 and 12 months of lactation. All subjects participating in the LIFE cohort had given written consent, and all procedures in this cohort obtained approval from relevant local authorities [14]. In the LIFE cohort, breast milk was obtained from non-fasted subjects with an electric breast pump (Medela, Baar, Switzerland) following a standardized collection protocol. The same procedure (including make and model of breast pump) and conditions were used in the small Swiss cohort.

### 4.3. Sample Pre-Treatment

Breast milk samples were centrifuged for 15 min at 21,000× *g* at 4 °C to remove fat and solids. The fat layer was compressed in a thin film at the surface. As its volume, in percentage of total milk volume, was considered not to exceed the typical percentage fat in a breast milk sample (~5%) and since folate is not fat-soluble, we considered skim milk folate values to reflect full milk values. Skimmed breast milk samples were subsequently diluted 7-fold with water, heated during 5 min at 90 °C, and then cooled on ice prior to analysis.

### 4.4. FOLA Method

The auto-analyzer Dimension^®^ ExL200™ with LOCI^®^ module, the FOLA kits, and the appropriate calibrator were all purchased from Siemens Healthineers (Zurich, Switzerland). The FOLA method is a homogeneous, competitive chemiluminescent immunoassay for quantification of all folate forms that can displace a folic acid derivative that is pre-bound to FBP. The method is certified for use with plasma samples, which are entered into the device without pre-treatment or dilution. As described in the present manuscript, folate measurement of breast milk samples with the FOLA method requires some sample pre-treatment and dilution and a correction of the readout by a dilution factor. At regular and recommended intervals the auto-analyzer was calibrated with plasma samples containing certified amounts of folate, spanning the linear range of the instrument.

### 4.5. Method Validation

The precision (repeatability and intermediate reproducibility), linearity, trueness, measurement uncertainty, and lower and upper limit of quantification (LLOQ and ULOQ) were determined with samples from the Swiss cohort. The samples had been diluted 7-fold and heat-treated as this was required to reduce matrix effects and ensure optimal recovery (see Section 2.1). After a first pass screening, a pool of 3 milk samples with the lowest apparent folate measures was chosen out of 10 women and set as the lower limit. This pool was then spiked with 5-MeTHF to yield 13.8, 34.5, 51.7, 68.9 and 103.4 nmol/L folate. Preparation of the spike solution is described in Section 4.1. Each pool sample was diluted, heated, and measured on 3 different days, in triplicate, each time by two different operators, resulting in 18 different measures per concentration.

For repeatability and intermediate reproducibility, standard deviations and coefficients of variation (CVs) were calculated. We aimed for CVs at all the levels to be <10% except for LLOQ for which a threshold of 15% was set.

Trueness was assessed using recovery. Recovery was calculated as R (%) = (Concentration C measured − C endogenous) × 100/C added. Recoveries were deemed acceptable if between 80%–120%. Uncertainty and relative uncertainty (see Table 2) were calculated based on the validation data using the bottom-up approach in accordance with Barwick and Ellison [25].

LLOQ and ULOQ were set as the lowest and highest concentration measured (8.4 nmol/L and 111.7 nmol/L, respectively) for which the performance, at least precision, was defined acceptable.

### 4.6. LC-MS/MS Measurement

Redeuil et al. recently described methodology for the quantification of several water soluble vitamers, including folate (5-MeTHF and folic acid), in human milk using reversed-phase chromatography combined with tandem mass spectrometry detection [10].

Briefly, 200 µL of full breast milk was spiked with 200 µL of α-amilase (0.25 mg/mL) and incubated at room temperature for 30 min. Internal standards were added (see Redeuil et al. for details [10]) as well as 600 μL methanol which contained 1% acetic acid. This was vortex mixed for 10 s and incubated for 5 min. at room temperature to allow proteins to precipitate. Proteins were pelleted by centrifugation at 21,000× *g* for 10 min. at 4 °C. The supernatant was evaporated to dryness under a mild stream of nitrogen in the dark. Dried extracts were subsequently reconstituted in 500 μL mobile phase “A” (water with 5% acetic acid, 0.2% heptafluorobutyric acid). Samples were filtered through a compatible filter with a cut-off of 0.45 μm before being subjected to reversed-phase chromatography. For the latter, an ACE-3 C18-pentafluorophenyl column (150 × 2.1 mm, 3 μm) was used in an Agilent 1290 Infinity system at a constant flow rate of 0.2 mL/min, using a gradient of mobile phase A and mobile phase B (pure acetonitrile). Finally, 10 μL of sample extract was injected into the LC-MS/MS system (6460 MS mass spectrometer using Jetstream mode applying positive ionization, Agilent, Santa Clara, CA, USA) 

### 4.7. Statistical Analyses

Statistical analysis during the method validation process was carried out according to European Medicines Agency standards (http://www.ema.europa.eu/pdfs/human/ewp/19221709en.pdf). Linearity was assessed by performing a linear regression of the average duplicates compared to the spike levels. The 95% confidence interval of the slope was compared to 1 and the coefficient of determination (R^2^) was also assessed. The same approach was adopted when comparing the FOLA method to the LC-MS/MS method. For precision, robust standard deviations (SDs) and coefficients of variation (CVs) were computed as described by Rousseeuw and Croux [26]. A Bland–Altman plot of FOLA vs. LC-MS/MS measurement is presented in Figure 2b. Bounds were calculated as bias + 1.96 times the standard deviation (SD) of the differences between methods.

## Figures and Tables

**Table 1:** Method performance characteristics at different spiking levels. CV, Coefficient of variation.

Spike Level (nM)	Median Measured Value (nM)	Repeatability	Intermediate Reproducibility	Recovery [%]	Uncertainty	Relative Uncertainty [%]
		CV [%]	CV [%]			
0	8.5	12.1	10.3	-	-	-
13.8	23.2	3.8	5.1	107	3.01	13
34.5	41.8	2.3	2.0	97	2.36	6
51.7	58.3	3.3	4.4	96	5.64	10
68.9	76.1	1.7	2.0	98	3.40	4
103.4	113.5	1.3	4.8	102	11.80	10

**Table 2:** (**a**) Folate levels in breast milk of German mothers at 3, 6, and 12 months of lactation. (**b**) Subject characteristics. At 3 and 6 months, 3 and 4 subjects respectively displayed folate levels above the upper limit of quantification and were thus not included in the table. n, number of subjects; SD, Standard deviation.

**a**			**Folate (nM)**				
	**Lactation Week**	**n**	**Average**	**SD**	**Median**	**Min**	**Max**
	12	243	27.2	15.8	23.4	6.2	89.6
	24	240	34.7	17.4	30.3	10.3	100.7
	52	66	29.5	19.2	24.1	9.0	97.9
**b**	**Maternal / infant parameters**				**n**		**Average (SD)**
	Maternal age at birth (years)				333		31.17 (4.55)
	Gestational age (weeks)				298		39.69 (1.67)
					**n**		**Percentage of Infants**
	Exclusive breastfeeding at 3 months				298		89%
					**n**		**Male/Female**
	Gender				333		175/158
	C-section births				324		57 (17.6%)

**Table 3:** Overview of reported values of breast milk folate in a variety of cohorts, determined with microbiology- or chromatography-based methodology.

Country, Year	n	Folate (nM)	Lactation Week	Method	Reference
Nigeria, 1980	30	11.3, 16.1	1, 2	Microbiology (Lactobacillus casei 6375), No pretreatment	[16]
Japan, 1980	31	320.4	3–25	Microbiology (L. casei), Conjugase pretreatment	[17]
USA, 1980	12	113.5	42	Microbiology (L. casei), Pretreatment unclear	[18]
USA, 1981	16	94.3, 97.0	1, 6–7	Microbiology (L. casei ATCC 7469), No pretreatment	[19]
UK, 1983	26–35	38.5, 70.2, 95.2	1, 2, 3–34	Microbiology (L. casei), Pretreatment unspecified	[20]
USA, 1986	55	97.4, 63.4, 90.6, 49.8	49, 84, 126, 168	Microbiology (L. casei ATCC 7469), No pretreatment	[21]
Canada, 1995	29	114.9, 124.6, 83.8	4, 8, 12	Microbiology (L. casei), Conjugase pretreatment	[22]
USA, 1998	84	205.3, 184.7	12, 24	Microbiology (L. casei), Trienzyme pretreatment.	[8]
USA, 1999	42	224.4, 187	12, 24	Microbiology (L. casei), Trienzyme pretreatment	[23]
Japan, 2005	4000	339.9, 231.1, 117.8, 131.4, 53.0	1, 2, 3–13, 14–25, 26- 52	HPLC	[12]
Mexico, 2006	71	103.1, 154.2, 143.3	3, 12, 20	Microbiology (L. casei), Trienzyme pretreatment	[24]
Canada, 2009	69 (3 groups)	181	16	Microbiology (L. rhamnosus ATCC 7649), Trienzyme pretreatment	[4]
USA, 2012	28	142.0 (microbiological), 86.1 (HPLC)	5–15	HPLC and Microbiology (L. casei ATCC 7469), Conjugase pretreatment.	[11]
Sweden, 2014	38	150	Unknown	HPLC	[13]
